# Supplementary material for: On the Mechanism of Membrane Permeabilization by Tamoxifen and 4-Hydroxytamoxifen
Source: Membranes (Basel). 2023 Feb 28;13(3):292. doi: 10.3390/membranes13030292 (PMC10058083; doi:10.3390/membranes13030292)
Supplement: Supplementary file 1 [file membranes-13-00292-s001.zip › membranes-2213098-supplementary.pdf]

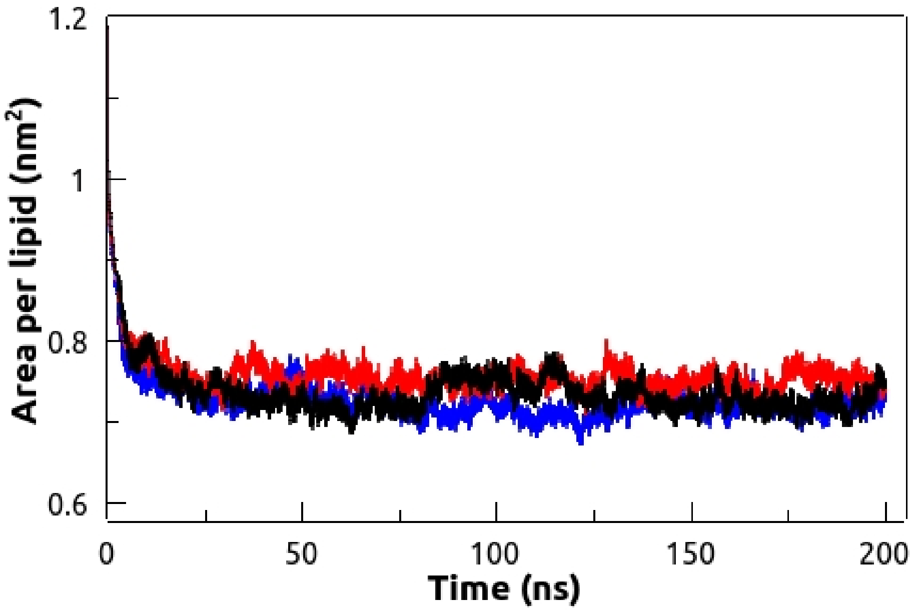

Figure S1. Area per lipid. Surface area per lipid vs. time of a single replica simulation of POPC/TMX (32:11) (black), POPC/POPE/TMX (24:8:11) (blue) and POPC/POPG/TMX (24:8:11) (red).

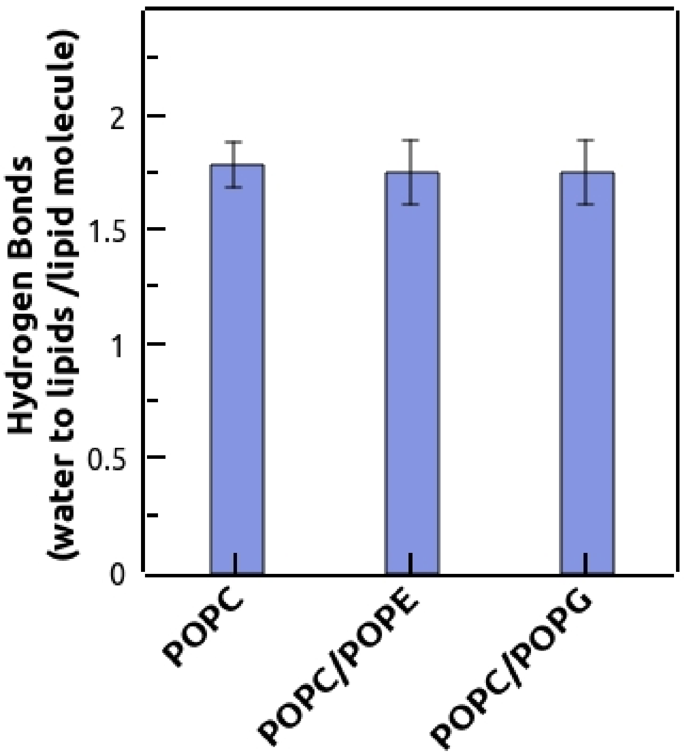

Figure S2. Carbonyl hydrogen bonding. Hydrogen bonds between water and phospholipid carbonyl groups in the different simulated phospholipid bilayers. Error bars represent the s.d. of the trajectories analyzed.

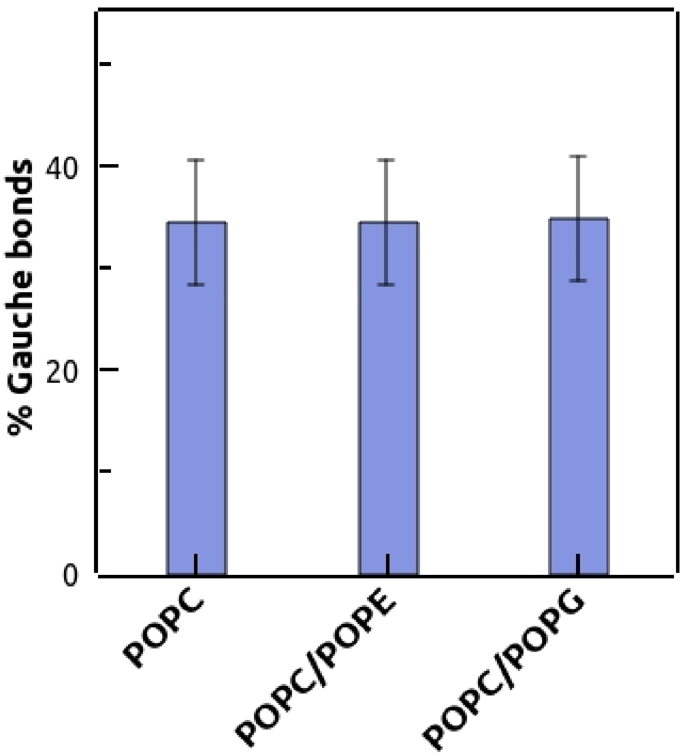

Figure S3. *Gauche* conformers. The proportion of *gauche* conformers in the different simulated phospholipid bilayers. Error bars represent the s.d. of the trajectories analyzed.
